# The Role of the Dissenting Voter on Jury Deliberation Online: A Pilot Study

**DOI:** 10.3390/bs16101960

**Published:** 2026-10-21

**Authors:** Tova Cohen, Colleen M. Berryessa

**Affiliations:** School of Criminal Justice, Rutgers University, Newark, NJ 07102, USA; colleen.berryessa@rutgers.edu

**Keywords:** jury deliberation, juror decision-making, conformity, social influence, conflict management, virtual juries, mock juries

## Abstract

Jury deliberation involves both individual judgments and interpersonal processes through which jurors exchange information, respond to disagreement, and work toward a collective decision. This mixed-methods pilot study examined individual characteristics, deliberative participation, verdict change, and dissent across seven virtual mock juries. Thirty-five participants took part in pre- and post-deliberation data collection and deliberated via Zoom in six-person juries that included a trained confederate operating under a conditional dissent protocol. Collaborative conflict-management style was positively associated with self-reported deliberative participation, whereas openness, agreeableness, competing conflict style, and civic engagement were not; however, the overall predictor model did not significantly improve fit relative to the unconditional model. Among 34 participants with complete verdict data, all six who initially selected not guilty changed to guilty following deliberation, producing a statistically significant paired shift that did not support the hypothesized direction of change. Confederate-introduced dissent occurred in six juries, while dissent emerged organically in one. Differences in deliberation length were therefore examined descriptively. Qualitative content analysis further indicated that exchanges involving dissent included substantive engagement with competing interpretations, evidentiary ambiguity, and jurors’ reasoning. These preliminary findings suggest that understanding jury decision-making requires attention not only to final verdicts, but also to how jurors participate in and respond to disagreement during deliberation.

## 1. Introduction

Although relatively few criminal cases are ultimately resolved through trial, jury deliberation remains an important component of the American criminal legal system and a consequential setting for collective legal decision-making. Jurors are asked to evaluate evidence independently, communicate their interpretations to others, and ultimately reach a collective decision with potentially substantial consequences for defendants and other parties involved. Legal doctrine presumes that this process promotes careful consideration of evidence and independent judgment (Legal Information Institute, 2023a, 2023b; Lerner & Thomas, 2015). However, decades of psychological research suggest that group decision-making may also expose individuals to conformity pressures and other forms of social influence that may hinder the goals of deliberation (Bergold & Bull Kovera, 2022; Davenport & Winet, 2022; Kruglanski et al., 2006; L. Torres, 2022; Waters & Hans, 2009). Understanding how jurors respond to disagreement and dissent, and how individual characteristics correspond with participation and collaborative engagement, is therefore important to understanding the deliberative processes underlying collective verdicts (Bartling et al., 2015; Davenport & Winet, 2022).

Jury deliberation serves a dual purpose: it exposes jurors to competing interpretations of the evidence while encouraging collective evaluation of the facts before a verdict is rendered (Hans, 2012). From this perspective, changing one’s opinion during deliberation is not inherently problematic. Instead, such a change may reflect thoughtful engagement with evidence, persuasive arguments, or alternative interpretations introduced by other jurors (Austen-Smith & Banks, 1996; Gastil et al., 2007). At the same time, social psychological research suggests that unanimity pressures, social desirability, and other normative influences in group settings may shape expressed preferences independently of sincere consideration of the evidence (Asch, 1951, 1956; Devine et al., 2001). Distinguishing genuine persuasion from social accommodation, then, remains an important challenge for jury deliberation research.

This distinction becomes particularly relevant when a juror encounters a clear majority position within their jury group. Research on group decision-making consistently demonstrates that individuals are susceptible to majority influence, especially in time-constrained and particularly consequential settings (Asch, 1951, 1957; Davenport & Winet, 2022; Kruglanski et al., 2006). Within juries, these pressures may be heightened by expectations of unanimity and the perceived consequences of continued disagreement, including prolonged deliberation or a hung jury (Barry, 2023; Curley et al., 2022; Davenport & Winet, 2022). Prior work suggests that jurors may alter their expressed preferences as the group moves toward consensus (Bergold & Bull Kovera, 2022; Gastil et al., 2007). It is important, however, to note that a change in verdict cannot by itself tell us whether a juror conformed to social pressure or genuinely reconsidered the evidence.

In this vein, the presence of a nonconforming opinion in a setting prone to conformity pressures provides a valuable opportunity to examine group decision-making. Minority influence research suggests that dissent can interrupt an emerging consensus, encourage deeper information processing, and prompt more thorough consideration of alternative perspectives (Asch, 1956; Bergold & Bull Kovera, 2022). However, the dissenter is also subject to group processes and may experience pressure to conform when their position conflicts with that of the larger group (Asch, 1951; Davenport & Winet, 2022; Kruglanski et al., 2006; L. Torres, 2022; Waters & Hans, 2009). Dissent, therefore, may matter even when it does not ultimately change the group’s verdict. Its importance may instead appear in the discussion itself: how jurors justify their positions, respond to disagreement, and consider perspectives that challenge an emerging consensus.

The influence of dissent may also depend on the individuals participating in the discussion. Jurors differ in their willingness to entertain alternative perspectives, in how they approach interpersonal conflict, and in how invested they are in collective decision-making. These differences may affect not only whether a juror reconsiders an initial position but also how they participate when disagreement emerges. Examining juror characteristics alongside group-level processes of dissent and social influence may therefore provide a more complete picture of how individual judgments develop through collective deliberation.

### 1.1. Juror Characteristics and Deliberative Engagement

Although jury deliberation is inherently a group process, jurors enter that process with individual characteristics that may shape how they participate, respond to disagreement, and consider competing interpretations of the evidence (Clark et al., 2007). Prior mock-jury research has examined the role of preexisting beliefs, attitudes, and personality characteristics in juror decision-making (Clark et al., 2007; Crocker & Kovera, 2011; Kovera & Borgida, 2010; Peter-Hagene, 2019; Wiener & Bornstein, 2011). Such differences may be especially relevant during deliberation, where jurors must do more than form an independent judgment. They must communicate that judgment, listen to others, and negotiate disagreements within a group setting.

Personality may provide one explanation for how jurors approach these interpersonal demands. Research drawing on the Five-Factor Model of Personality has linked personality characteristics to differences in judgment, openness to information, and interpersonal behavior (Costa & Widiger, 2002). Of particular interest to the present study are openness and agreeableness. Openness reflects receptivity to new ideas, experiences, and alternative perspectives and may, therefore, make jurors more willing to consider competing interpretations during discussion. Indeed, open-mindedness has been identified as a favorable juror characteristic because it can encourage thoughtful consideration of information and perspectives that differ from one’s initial judgment (Olczak et al., 1991; Sprain & Ivancic, 2017). Agreeableness, on the other hand, reflects interpersonal tendencies toward cooperation and consideration of others and may similarly facilitate constructive engagement when disagreement arises (Lindell & Strandberg, 2025).

Personality traits alone, however, may not fully explain how individuals behave once disagreement actually occurs. Conflict management styles provide a more interaction-focused framework for understanding how group members respond to competing preferences and perspectives (Kuhn & Poole, 2000; Nguyen et al., 2019). Individuals may approach conflict through strategies characterized by varying levels of cooperation and assertiveness, including accommodating, avoiding, competing, and collaborating approaches (Nguyen et al., 2019). These approaches are particularly relevant to jury deliberation, where jurors are expected to reconcile individual judgments while still working toward a collective resolution.

Collaborative conflict management, specifically, emphasizes active engagement with disagreement and attempts to develop a resolution that considers the concerns or perspectives of multiple group members (Adkins, n.d.). Such an approach may be particularly conducive to deliberation because it encourages jurors to explain their reasoning, consider opposing interpretations, and remain engaged when conflict emerges (Gordon, 2014). Prior group research suggests that compatible and collaborative approaches can facilitate a common resolution, whereas competing or avoidant approaches may yield outcomes less consistent with individual preferences (Nguyen et al., 2019). In the jury context, collaborative conflict management may be especially important because it encourages substantive interaction rather than withdrawal from or domination of disagreement (Gordon, 2014).

Finally, jurors may differ in the extent to which they view deliberation as a meaningful form of civic participation. Jury service provides lay citizens with a direct role in legal decision-making and has been linked to broader forms of civic engagement, including later voting and volunteering (Gastil et al., 2007, 2010; L. Pennington & Dolliver, 2021). Research on civic participation also emphasizes the role of intrinsic motivation in sustaining engagement in collective activities (Dolliver, 2024). Jurors who are more civically engaged may therefore approach jury service with a greater sense of investment, responsibility, or willingness to participate in the deliberative process.

Overall, this literature suggests that deliberative engagement may reflect both relatively stable individual characteristics and the ways jurors approach interpersonal disagreement. Openness and agreeableness may encourage receptivity and cooperative interaction, collaborative conflict management may promote active engagement with competing viewpoints, and civic engagement may increase investment in the deliberative task itself. Evaluating these characteristics alongside group-level deliberative processes allows the present study to consider both who participates in deliberation and how disagreement unfolds once discussion begins.

### 1.2. Group Decision-Making, Dissent, and Verdict Change

Jury deliberation requires individuals to reconcile their own judgments with the perspectives expressed by other group members. This process creates opportunities to reconsider evidence and refine interpretations, but it also exposes jurors to informational and normative forms of social influence. As a jury moves toward consensus, those holding minority positions may face increasing pressure to reconsider or publicly modify their initial preferences (Davenport & Winet, 2022; Devine et al., 2001). Comparing jurors’ initial preferences with their post-deliberation verdicts, therefore, provides one way to examine how individual judgments develop through collective discussion.

Social psychological research demonstrates that majority consensus can influence expressed judgments. Asch’s (1951, 1956, 1957) conformity studies found that individuals sometimes aligned their public judgments with an incorrect majority, although this influence was substantially reduced when unanimity was disrupted by even one dissenting group member. Research applying these principles to jury decision-making similarly suggests that unanimity requirements and prevailing voting distributions can shape group behavior (Davenport & Winet, 2022; Garcia et al., 2021). Research with actual jurors further demonstrates that some individuals ultimately vote with the majority despite privately preferring a different verdict (Waters & Hans, 2009). These findings highlight the potential influence of group consensus, particularly when judgments are expressed publicly rather than privately (Asch, 1957).

However, changes in verdict preferences following deliberation should not automatically be interpreted as evidence of conformity. Deliberation facilitates the exchange and collective evaluation of information, and jurors may reconsider their initial judgments when others identify overlooked evidence, offer persuasive interpretations, or construct more coherent accounts of the case (Gastil et al., 2007; N. Pennington & Hastie, 1992; Salerno & Diamond, 2010). Movement toward consensus may therefore reflect genuine evidentiary reconsideration, normative social accommodation, or some combination of the two. Without directly assessing the mechanisms underlying verdict changes, pre- and post-deliberation preferences alone cannot distinguish between these explanations.

Dissent provides a useful context for examining these processes. Jurors expressing positions that differ from an emerging majority may encourage further discussion by prompting others to justify their reasoning, revisit overlooked evidence, or consider alternative interpretations (Asch, 1956; Bergold & Bull Kovera, 2022; Sprain & Ivancic, 2017). At the same time, dissenting jurors may experience pressure to align with the majority as discussion progresses and expectations of unanimity strengthen (Davenport & Winet, 2022; Waters & Hans, 2009). Consequently, the significance of dissent may extend beyond its influence on the final verdict to include how jurors engage with competing perspectives throughout deliberation.

Importantly, dissent may also differ according to how it emerges within the group. Disagreement may arise organically when a group member independently expresses a position that conflicts with an emerging consensus, or a dissenting perspective may be deliberately introduced to challenge an otherwise convergent group. Prior research suggests that these distinctions may be consequential for group decision-making. Nemeth et al. (2001) found that authentic minority dissent was more effective than assigned devil’s advocacy in stimulating divergent thinking and generating alternative solutions. Similarly, Schulz-Hardt et al. (2002) found that genuine disagreement was more effective than deliberately introduced devil’s advocacy in reducing confirmatory information-seeking biases. Related research suggests that disagreement among group members before discussion can facilitate information exchange and increase discussion intensity (Schulz-Hardt et al., 2006).

Together, these findings suggest that the emergence and source of dissent may shape how groups evaluate competing perspectives and engage in collective decision-making. However, experimentally assigned devil’s advocacy differs from the conditional confederate protocol employed in the present study, and findings from these studies cannot be assumed to generalize directly to mock-jury deliberation. Nevertheless, distinguishing organically emerging disagreement from confederate-introduced dissent offers an opportunity to examine whether these forms of disagreement correspond to different patterns of deliberative engagement, including the time required to reach a collective decision.

Examining individual verdict changes alongside dissent and deliberative participation allows the present study to consider both the outcomes of deliberation and the interpersonal processes through which those outcomes develop. Rather than presuming that post-deliberation changes constitute conformity, this approach considers whether individual judgments shift following group discussion and how jurors respond when disagreement enters the deliberative process.

### 1.3. Virtual Deliberation Context

The increased use of remote legal proceedings since the global COVID-19 pandemic has generated growing interest in how jury deliberation operates in technologically mediated environments (Hans, 2021; Reed et al., 2026; L. C. Torres et al., 2024). Although early virtual proceedings primarily involved pretrial hearings and civil matters, courts subsequently expanded the use of remote procedures across other stages of the jury process (Hans, 2021). These developments have raised questions about whether virtual environments alter how jurors attend to information, interact with one another, and engage in collective decision-making.

Existing research suggests a relatively nuanced picture. Studies have generally identified few substantive differences between virtual and in-person juries in demographic composition or overall deliberative quality (Hans, 2021; Reed et al., 2026). At the same time, virtual formats raise concerns regarding attentiveness and engagement because videoconferencing fatigue, technological difficulties, and distractions in participants’ home environments may create additional opportunities for disengagement (Abrams, 2022; Hans, 2021; Pasnick, 2024). More recent work, however, suggests that although virtual participation may require greater cognitive exertion, videoconferencing fatigue does not necessarily translate into poorer deliberation (Reed et al., 2026). Similarly, research examining trial modality suggests that differences in presentation format may have relatively limited effects on ultimate verdict decisions while still shaping jurors’ perceptions, emotional responses, or information processing (Maeder et al., 2024; Reed et al., 2026). Taken together, this literature suggests that core decision outcomes and aspects of deliberative quality may be relatively robust across modalities, even as the processes through which jurors interact with information and one another may differ.

Comparatively less is known about how interpersonal processes such as disagreement, participation, and collective decision-making unfold during virtual jury deliberation. The present mock-jury study was conducted via videoconference to examine these relational processes in an online setting. Importantly, however, no in-person comparison condition was included in the current pilot study, meaning that deliberation modality was not manipulated. The virtual format is therefore treated as the context of the present study rather than as a causal explanation for the patterns observed.

### 1.4. Current Study

The present pilot study examines individual and group processes associated with deliberative engagement and decision-making in virtual mock juries. Specifically, the study considers whether personality, conflict management style, and civic engagement correspond with juror participation; whether individual verdict preferences change after group deliberation; and how nonconforming opinions emerge and are addressed during discussion. To provide additional context for individual verdict decisions, the post-deliberation survey included exploratory items assessing when participants reported reaching their verdict and the perceived influence of case evidence and group discussion. These retrospective self-reports offer preliminary insight into participants’ perceptions of their decision-making processes, but cannot establish the mechanisms responsible for verdict changes.

Thirty-five participants deliberated across seven six-person mock juries conducted via Zoom, with each group including one trained confederate who was excluded from the participant count. Her role depended on the opinions expressed before her turn; if the five non-confederate jurors converged on the same position, she was instructed to cast a dissenting vote, using case-relevant information to support her divergent opinion. If disagreement among jurors emerged organically, the confederate could then participate independently rather than serving as the designated dissenter. Thus, the present design utilizes a conditional dissent protocol rather than a randomized confederate presence-versus-absence manipulation.

Based on the extant literature, our study team developed three hypotheses.

First, individual characteristics were expected to correspond with jurors’ engagement in the deliberative process (**H1**):

**H1a.**  *Jurors reporting greater openness and agreeableness are expected to exhibit greater deliberative engagement, including greater willingness to consider opposing viewpoints and participate substantively in group discussion.*

**H1b.**  *Jurors reporting more collaborative conflict-management styles are more likely to exhibit greater deliberative engagement and participation during deliberation.*

**H1c.**  *Jurors reporting greater civic engagement are expected to demonstrate greater deliberative participation and perceived investment in the deliberative process.*

Second, consistent with research demonstrating that individual judgments may change through collective discussion, the study examined whether jurors’ verdict preferences differed before and after deliberation:

**H2.**  *Jurors exposed to dissent during group deliberation are significantly more likely to shift their post-deliberation verdict preferences toward the confederate’s vote compared to their pre-deliberation baseline assessments.*

Grounded in foundational research on group influence and independence (Asch, 1951, 1956, 1957), encountering nonconforming opinions during group discussion disrupts the pressure toward unanimity and encourages independent re-evaluation of judgments. Because the dissenting confederate introduced counterarguments highlighting reasonable doubt, we predict a directional shift in individual verdict preferences toward the confederate vote following deliberation, rather than an unguided or arbitrary change.

Finally, given that the source of dissent may shape how groups work through disagreement, we examined whether deliberation duration differed according to how dissent emerged:

**H3.**  *Deliberation length will differ between juries in which dissent emerges organically and juries in which the confederate introduces a dissenting position.*

## 2. Materials and Methods

### 2.1. Study Design

As stated above, the present pilot study employed an online mixed-methods simulated-jury design to examine individual characteristics, deliberative participation, verdict change, and structured dissent during virtual jury deliberations. After providing informed consent, participants individually reviewed the criminal case vignette and jury instructions (See Appendix A). They then completed an individual pre-deliberation survey (Survey 1) assessing personality traits, conflict management styles, civic engagement, and initial verdict preference based on the case materials. Following Survey 1, the case vignette and jury instructions were presented again to the group and read aloud by the experimenter before participants began deliberating in six-person mock jury groups. One trained confederate was included in each group, with her role depending on whether unanimity had emerged among the five non-confederate jurors before her turn. Following deliberation, participants completed an individual post-deliberation survey (Survey 2) assessing final verdict preference and perceptions of the deliberative process. The recorded discussions were also observationally coded to capture patterns of juror argumentation and participation.

### 2.2. Participants

Participants were undergraduate and graduate students recruited in person and via email from large introductory courses and through snowball sampling at a large state university in the southeastern United States. Participants were invited to take part in an approximately one-hour virtual mock-jury session conducted via Zoom 5. 13. 10. Depending on course arrangements, participants received either course extra credit or a $20 gift card as compensation for their participation. Deliberation sessions were conducted only when six individuals were present; sessions with insufficient attendance were rescheduled for a later date and time. The confederate was not included in the analytic participant sample and did not complete the study surveys. Across seven deliberation groups, the final analytic sample therefore consisted of 35 participants. Participant demographics are reported in Table 1.

Two trained confederates were used across data collection to promote consistency in the implementation of the study protocol. Both were female students aged 19 to 23. Confederates received standardized instructions, a script, and a checklist outlining how to respond to different distributions of juror opinions during deliberation. The checklist included anticipated arguments, potentially overlooked details from the case materials, and space to record arguments raised before the confederate’s turn so that the confederate could respond appropriately. Additional confederate prompts and procedural materials are provided in the Supplementary Materials. Both confederates completed joint training using standardized scripts and response matrices to handle participant inquiries uniformly. Protocol adherence was maintained across sessions through post-deliberation compliance checklists completed by the experimenter, ensuring consistent execution across both confederates.

### 2.3. Materials and Procedure

Upon entering the Zoom session, participants and the confederate were assigned anonymized labels (e.g., “Juror #1”) to both enhance subject confidentiality and simulate a more realistic jury experience. After providing informed consent, participants completed an individual pre-deliberation survey. The survey included adapted items from the Big Five Inventory–2 Short Form (BFI-2-S; Soto & John, 2017), the Conflict Management Styles Assessment (Adkins, n.d.), and civic engagement items drawn from the Survey of Texas Adults (Musick, 2004), as well as participants’ initial individual verdict preferences based on case material and jury instructions (see Appendix A and Appendix B).

Upon completion of Survey 1, the case vignette and jury instructions were presented again to the full group. The materials were distributed electronically and read aloud by the experimenter before the recorded deliberation began. Participants were informed that the deliberation would be audio- and video-recorded for coding purposes. This second presentation ensured that all jurors entered the group discussion having fully read and understood their task. A case involving a violent sexual assault of a minor was selected because the defendant’s alleged failure to report suspected abuse created ambiguity regarding individual culpability despite the seriousness of the underlying conduct, providing a context in which jurors could plausibly differ in their interpretations of the defendant’s legal responsibility. Given the graphic nature of the case materials, participants also received a content warning and were provided with support resources at the conclusion of the study. The vignette was developed using information from the national survey of mandated reporters (Zellman, 1992) and with consultation from an expert with a background in criminology and law.

Participants also received standardized criminal jury instructions (see Appendix B) adapted and abridged from federal criminal jury materials (Baguley et al., 2020; Legal Information Institute, 2023a). Preliminary pilot sessions indicated that the full instructions were overly lengthy for the simulated deliberation procedure. The vignette and jury instructions were distributed electronically and read aloud by the experimenter before recorded deliberation began. One participant, excluding the confederate, served as foreperson, or deliberation facilitator, of the group. The foreperson either volunteered for the role or was selected through random assignment. During deliberation, the experimenter did not participate in the group discussion and reappeared only after the jury reached a verdict or determined it could not reach one. Jurors were then asked to state their verdicts aloud, with the confederate called upon last each time.

Following deliberation, participants completed a post-deliberation survey (Survey 2) assessing final verdict preference and perceptions of the deliberative process. Deliberation items, adapted from the Deliberation Issues Scale (Waters & Hans, 2009), assessed aspects of participation quality, collaborative interaction, openness to differing perspectives, and opportunities to express disagreement. Example items included “How thoroughly was each juror’s point of view considered?” and “How open-minded was the jury to each other’s ideas?” Pre- and post-deliberation responses were linked using self-generated identification codes (Kearney et al., 1984; Little et al., 2022). Participants were then debriefed and dismissed.

### 2.4. Confederate Dissent Protocol

**Figure 1:**
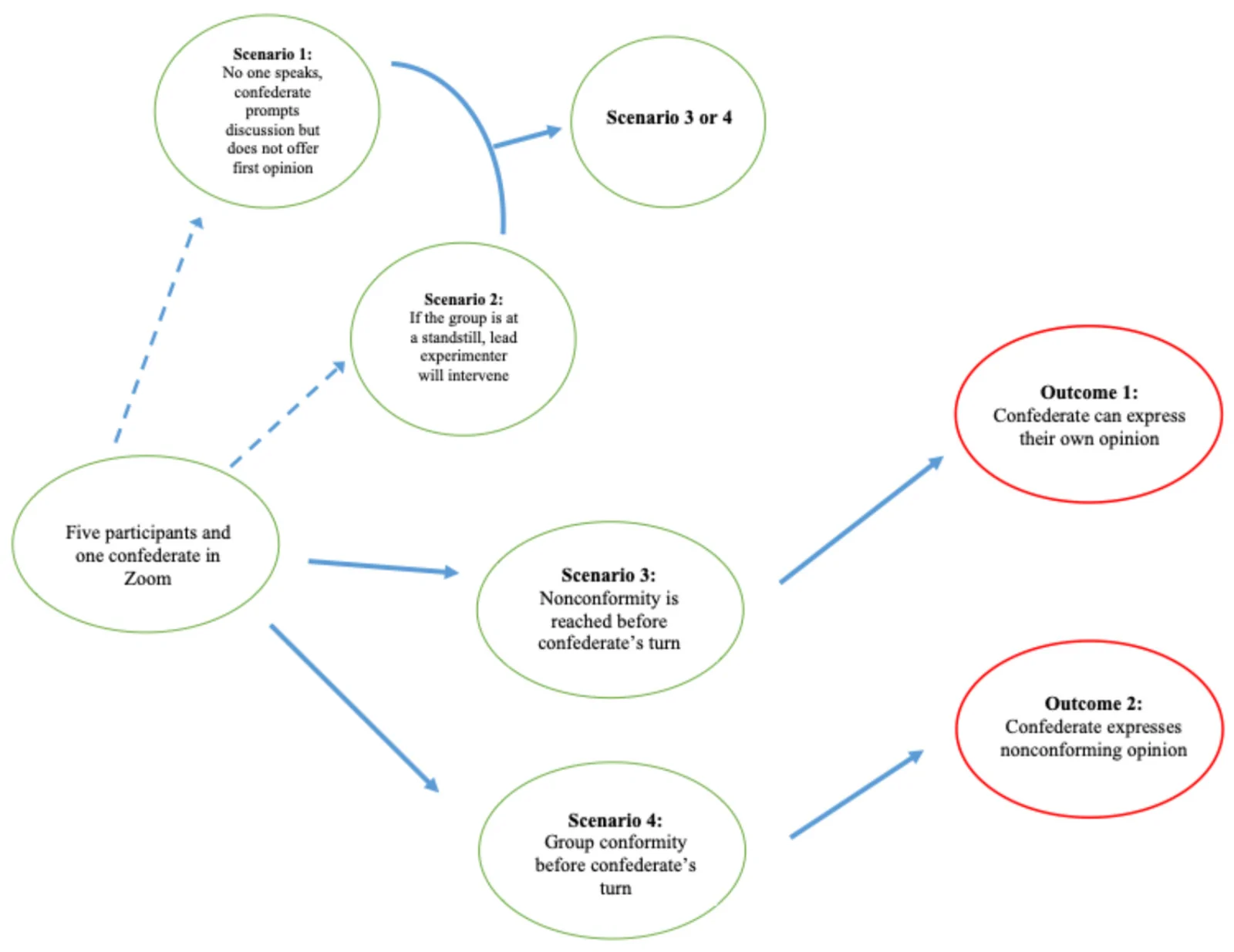
The Trajectory of the Confederates’ Voting Behavior.

Each deliberation group included one trained confederate positioned to respond to myriad case-related and emotionally charged points raised by the other jurors. While a confederate was physically present in every group, her acting role as the designated dissenter depended on whether group unanimity had emerged before her turn to state a verdict, as visualized in Figure 1. When all five non-confederate jurors had expressed the same verdict preference before the confederate’s turn, the confederate cast an opposing vote and introduced an alternative interpretation of the evidence supported by case-relevant reasoning. The purpose of this procedure was to ensure that a nonconforming perspective entered the discussion after an otherwise dominant-group position had developed, rather than to require the confederate to persuade the group or to prevent an eventual unanimous verdict. Following subsequent discussion, the confederate could ultimately align with the group’s position unless other jurors continued to express an opposing preference.

If group unanimity had yet to emerge by the confederate’s turn, the confederate was not required to adopt a dissenting position and instead participated in accordance with personal preference. If no unanimous decision emerged within the first ten minutes, the confederate was instructed to use a neutral discussion prompt to get the conversation moving. Both confederates followed a standardized script and checklist while retaining sufficient flexibility to respond organically to arguments raised by participants.

For consistency with the study’s original coding scheme, sessions in which the confederate enacted the designated dissenting role were coded as “confederate dissent present,” whereas sessions in which she voted independently because no unanimous position had emerged were coded as “confederate dissent absent.” Because enactment of the dissenting role depended on the naturally developing distribution of jurors’ verdict preferences rather than random assignment, this condition should not be interpreted as a randomized experimental manipulation of dissent.

### 2.5. Measures

Personality was assessed using the 30-item Big Five Inventory–2 Short Form (BFI-2-S; Soto & John, 2017). Participants rated the extent to which each characteristic described them on a five-point Likert scale ranging from 1 (disagree strongly) to 5 (agree strongly). Consistent with the study hypotheses, analyses focused on the Agreeableness and Open-Mindedness domains. Each domain was assessed using six items in accordance with the published BFI-2-S scoring procedure. Negatively keyed items were reverse-scored, and responses were averaged within each domain, with higher scores indicating greater levels of the corresponding personality characteristic. Internal consistency was acceptable in the present sample for Agreeableness (α = 0.78) and Open-Mindedness (α = 0.77).

Conflict management style was assessed using the 15-item Conflict Management Styles Assessment provided by the University of North Florida (UNF), which attributes the adaptation to Dr. Reginald Adkins and is based on the Thomas–Kilmann model. Participants indicated how frequently they had engaged in each conflict-management behavior during the previous six months using a five-point scale ranging from 1 (rarely) to 5 (always). The assessment measures five conflict-management styles: competing, collaborating, avoiding, accommodating, and compromising. Consistent with the study’s hypotheses, the present analyses focused on collaboration and competition. Each style was assessed using three items, which were averaged to retain the original 1-to-5 response metric; higher scores indicated greater use of the corresponding conflict-management style. Internal consistency in the present sample was acceptable for the collaborating (alpha = 0.76) and competing (alpha = 0.70) factors.

Civic engagement was assessed using a composite index adapted from the Survey of Texas Adults (Musick, 2004). The measure comprised 13 items spanning behavioral participation, attitudes toward legal institutions, and civic obligations. Binary behavioral items—voting history, contacting government officials, protest participation, jury summons, and prior jury service—were coded 0 (*No/Uncertain/Ineligible*) or 1 (*Yes*). News consumption frequency was scored on a 5-point scale from 0 (*Never*) to 4 (*Everyday*). Willingness to serve on a jury was measured on a 7-point Likert scale ranging from 1 (*Not at all willing*) to 7 (*Very willing*). Four items evaluated institutional preferences across legal scenarios (e.g., accuracy, criminal defense, civil lawsuits), with responses coded as 1 for selecting a jury and 0 for selecting a judge or indicating no preference. Finally, perceived civic duties (voting and community volunteering) were each rated on 4-point scales from 0 (*Not at all important*) to 3 (*Very important*). Individual item scores were summed to create an overall composite index with a theoretical range of 1 to 26, where higher scores reflected greater civic participation and orientation. In the current sample, observed scores ranged from 7 to 26 (M = 18.66, SD = 3.83). Missing data were handled using listwise deletion; participants with incomplete responses on the civic engagement index were excluded from relevant primary analyses.

Following deliberation, participants completed items adapted from Waters and Hans (2009) assessing multiple aspects of the deliberative process, including group openness, individual participation and influence, dominance by other jurors, consideration of differing viewpoints, perceived conflict, and adequacy of decision time. Because these items assessed conceptually distinct features of deliberation rather than a one-dimensional construct, they were treated as individual indicators rather than combined into a single scale. The primary measure of deliberative participation asked subjects how much they participated in the decision-making process, with responses ranging from 0 (none at all) to 3 (a great deal). Additional items were used to characterize participants’ perceptions of the broader deliberative process.

Participants reported an individual verdict before (Survey 1) and after jury deliberation (Survey 2). Pre-deliberation verdict preferences were collected as part of Survey 1 after participants had individually reviewed the case vignette and jury instructions, but before the materials were presented again to the group and before deliberation began. Participants indicated whether they believed the defendant was “Guilty” or “Not Guilty.” Post-deliberation verdict preferences were collected individually in Survey 2 immediately following group deliberation using the same response options. Verdict responses were coded dichotomously as guilty or not guilty. Changes between pre- and post-deliberation verdicts were used to assess whether individual verdict preferences shifted following group discussion.

The post-deliberation survey also included three exploratory items assessing participants’ perceptions of their verdict decision-making. Two items assessed the perceived influence of case evidence and group discussion, respectively, using four response categories ranging from 0 (not at all influential) to 3 (very influential). A third item assessed when participants reached their verdict, with response options including when they first read the case materials, after the first person spoke, during group discussion, after the last person spoke, and while completing the post-deliberation survey. These items were analyzed descriptively to provide additional context for the observed verdict transitions rather than to identify causal mechanisms of opinion change.

### 2.6. Quantitative Analytic Strategy

Because participants deliberated in seven separate mock juries, the observations may not have been independent. H1 was therefore evaluated using a hierarchical linear model (HLM) with participants nested within juries and a random intercept for jury number. Open-Mindedness, Agreeableness, collaborating and competing conflict-management styles, and civic engagement were entered simultaneously as individual-level predictors of self-reported participation. Given that the sample included only seven jury-level clusters (*J =* 7), these multilevel estimates are treated cautiously, as they are exploratory.

H2 was evaluated by tracking individual paired pre- and post-deliberation verdict transitions among participants with complete data at both timepoints (*n* = 34). To examine individual-level shifts, an exact McNemar test was conducted. However, because participants deliberated in seven clustered jury groups (*J* = 7), individual vote changes are nested within juries and subject to non-independence. Given the small number of jury clusters (*J* = 7) and the small number of discordant cells, complex multi-level binary logistic models or clustered McNemar extensions (Eliasziw & Donner, 1991) could not be reliably estimated. Consequently, the McNemar test is interpreted with caution as a descriptive summary of paired individual transitions rather than as a strict inferential test of independence. H3 was examined descriptively at the jury level by comparing deliberation duration across juries in which dissent emerged organically and those in which the confederate introduced a dissenting position. Because organic dissent occurred in only one jury, an inferential comparison of deliberation length by dissent source was not conducted.

### 2.7. Observational and Content Analysis

To capture features of deliberation not fully represented by the survey measures, all seven recorded deliberations were also examined using an inductive content analysis. The first author independently read each transcript in its entirety and identified recurring themes and subcategories rather than applying a predetermined thematic framework (Abukmail et al., 2025; Scott et al., 2019). The resulting coding focused broadly on two dimensions of deliberation: argumentation patterns and participation dynamics. Original coding notes distinguished emotional and factual reasoning, foreperson tone and participation, and confederate tone and participation. Argumentation patterns captured the ways jurors justified, questioned, or defended their verdict positions. Evidence-based reasoning primarily referred to arguments grounded in the vignette, jury instructions, mandated reporting responsibilities, and interpretations of reasonable suspicion or legal responsibility. Emotionally or morally oriented reasoning captured arguments that emphasized moral discomfort, perceived irresponsibility, the child’s vulnerability, the seriousness of the outcome, or broader expectations about how a responsible professional should have acted. Importantly, these categories were not treated as mutually exclusive, as the same juror or discussion could draw on both forms of reasoning simultaneously.

Participation dynamics, on the other hand, captured how jurors entered and sustained discussion, responded to disagreement, and moved toward consensus. Particular attention was paid to foreperson facilitation, confederate participation, responses to dissent, and moments in which jurors acknowledged ambiguity or entertained an alternative interpretation of the evidence. Engagement with an alternative perspective required substantive acknowledgment, consideration, or response to reasoning that differed from a juror’s previously expressed position; merely repeating the opposing argument was not considered sufficient. The transcripts and recordings of these focus groups were also used to code jury-level process characteristics, including whether dissent emerged organically from a non-confederate juror, whether the confederate introduced a dissenting position, the duration of deliberation, and the final jury verdict. Organic dissent was defined as an explicitly dissenting verdict or position expressed by a non-confederate juror before the confederate cast the last vote in the initial voting sequence. The confederate protocol specified that when early agreement emerged without substantive disagreement, the confederate would introduce a case-relevant opposing interpretation; when disagreement emerged organically, the confederate could participate as a normal juror.

All qualitative coding was conducted independently by the first author. Because a second coder was not used, there were no coder disagreements to reconcile, and interrater reliability was not assessed. The qualitative findings should therefore be read as exploratory descriptions of recurring patterns within these seven deliberations rather than as independently validated thematic classifications.

## 3. Results

### 3.1. Quantitative Results

Descriptive statistics for the study variables are presented in Table 2. Participants reported relatively high levels of Open-Mindedness (M = 3.48, SD = 0.76), Agreeableness (M = 3.95, SD = 0.65), and collaborative conflict management (M = 3.86, SD = 0.70). Mean competing conflict-management style was 3.32 (SD = 0.78), while the mean civic-engagement score was 18.66 (SD = 3.83). Participants reported an average deliberative-participation score of 1.74 (SD = 0.70).

For Hypothesis 1, a hierarchical linear model (HLM) examined whether Open-Mindedness, Agreeableness, competing and collaborating conflict-management styles, and civic engagement were associated with participants’ self-reported participation during deliberation. As shown in Table 3, neither Open-Mindedness (b = −0.003, SE = 0.150, *p* = 0.983) nor Agreeableness (b = −0.098, SE = 0.214, *p* = 0.649) was significantly associated with participation, failing to support H1a. Collaborating conflict-management style was positively associated with participation (b = 0.398, SE = 0.181, 95% CI [0.043, 0.753], *p* = 0.035), whereas competing conflict-management style was not (b = 0.078, SE = 0.165, *p* = 0.638). Thus, H1b received partial support. Although the individual coefficient for collaborative conflict-management style reached statistical significance, the full predictor model did not significantly improve overall model fit relative to the unconditional model, and the estimated random-intercept variance was zero. Consequently, this finding should be interpreted as exploratory and preliminary. Civic engagement was also unrelated to participation (b = −0.038, SE = 0.029, *p* = 0.188), failing to support H1c. The estimated jury-level variance was zero (ICC = 0.00), resulting in a singular model fit. Further, the full predictor model did not significantly improve fit relative to the unconditional model, χ^2^(5) = 6.88, *p* = 0.230. Given the small number of jury-level clusters and singular model fit, these estimates should be interpreted with caution.

For Hypothesis 2, initial verdict preferences were available for all 35 participants, of whom 29 (82.9%) selected guilty and six (17.1%) selected not guilty. Post-deliberation verdict preferences were available for 34 participants, as one participant in Jury 5 did not provide a post-deliberation response. Among the 34 participants with complete paired data, 28 (82.4%) initially selected guilty, and six (17.6%) selected not guilty. Following deliberation, all 34 participants reported guilty verdict preferences. Thus, all six participants who initially selected not guilty changed their verdict to guilty, while all 28 participants who initially selected guilty maintained that position. An exact McNemar test indicated a significant difference between paired pre- and post-deliberation verdict responses (*p* = 0.031; see Table 4 and Table 5). Although this finding indicates a change in verdict preferences following deliberation, participants did not shift toward the confederate’s dissenting position. Instead, all observed changes were from not guilty to guilty, reflecting movement toward the prevailing majority position.

Furthermore, because individual jurors were nested within seven deliberating juries, their vote shifts were not independent. The jury-level distribution of verdict transitions (Table 5) indicates that the six individual verdict shifts occurred across five of the seven juries. Jury 3 exhibited two shifts from not guilty to guilty, while Juries 2, 4, 5, and 7 each exhibited one shift. No individual verdict shifts occurred in Juries 1 or 6. One participant in Jury 5 did not provide a post-deliberation verdict, resulting in 34 complete pre- and post-deliberation responses across the seven juries. Given the clustering of individual responses within deliberating groups, the McNemar test result should be interpreted cautiously. These findings descriptively demonstrate movement toward unanimous guilty verdict preferences following deliberation, but cannot establish whether the observed changes reflected conformity pressures, reconsideration of the evidence, or other deliberative influences.

To further contextualize individual verdict preferences, participants reported the perceived influence of case evidence and group discussion on their verdicts, as well as when they reached them. Of the 35 participants, 32 (91.4%) rated case evidence as very influential, while three (8.6%) rated it as somewhat influential. Perceptions of group discussion were more varied. Among the 33 participants who responded to this item, 14 (42.4%) rated group discussion as very influential, 11 (33.3%) as somewhat influential, five (15.2%) as not super influential, and three (9.1%) as not at all influential. Regarding decision timing, 27 participants (77.1%) reported reaching their verdict when they first read the case materials, five (14.3%) after the first person spoke, two (5.7%) during group discussion, and one (2.9%) after the last person spoke. No participants reported reaching their verdict while completing the post-deliberation survey. These descriptive findings suggest that participants generally perceived the case evidence as highly influential, while the perceived influence of group discussion varied. However, these retrospective self-reports cannot establish the mechanisms underlying changes in individual verdicts.

As further shown in Table 5, Hypothesis 3 predicted that deliberation length would differ between juries in which dissent emerged organically and juries in which the confederate introduced a dissenting position. Of the seven juries, confederate-introduced dissent occurred in six (85.7%), whereas organic dissent occurred in only one (14.3%). Confederate-introduced dissent juries deliberated between 6 and 12 min (M = 9.17), while the organically dissenting jury deliberated for 16 min, making it the longest deliberation observed. Given that organic dissent occurred in only a single jury (*n* = 1), comparisons with juries involving confederate-introduced dissent (*n* = 6) are strictly descriptive and highly limited. While the single organically dissenting jury exhibited the longest observed deliberation time in the sample, no conclusions or generalizations regarding differences by source of dissent can be drawn from this pattern.

### 3.2. Observational and Content Analyses Results

The content analysis revealed two broad, recurring dimensions of group discussion: argumentation patterns and participation dynamics. Across the seven juries, differences were less apparent in the final jury-level outcome—all groups ultimately reached a guilty verdict—and more visible in how jurors justified their positions, responded to disagreement, and negotiated ambiguity throughout discussion.

When examining argumentation patterns, jurors commonly relied on both evidence-based/legal reasoning and emotionally or morally oriented reasoning. Evidence-based arguments centered on the therapist’s status as a mandated reporter, whether the circumstances provided reasonable suspicion of abuse, the former patient’s history, the child’s appearance and behavior, and the consequences of making a good-faith report. For instance, during Jury 2’s deliberations, one juror emphasized that the therapist was actively working in a professional capacity, had shown some degree of suspicion by approaching the former patient, and therefore had a responsibility to report the situation, which contributed to their guilty vote. Emotionally and morally oriented arguments, by contrast, emphasized the child’s vulnerability, the severity of the eventual harm, and judgments about the therapist’s professional or moral responsibility. During Jury 7’s discussion, for example, the foreperson emphasized that the child’s death made the therapist’s failure to report substantially more serious and questioned whether the therapist should remain in the profession after failing to act. Later in the same discussion, the same participant framed intervention as something a person with “good morals” should have done.

Interestingly, these two forms of reasoning often overlapped. Jurors invoked legal responsibility and factual details while also drawing on moral judgments, personal experience, or hypothetical assumptions. The transcripts, therefore, do not support a simple division between jurors who reasoned factually and those who reasoned emotionally. Rather, jurors frequently integrated both forms of reasoning when constructing and defending their verdict preferences.

### 3.3. Participation Dynamics and Engagement with Dissent

Participation also varied in both frequency and function. Forepersons commonly structured the discussion by collecting initial verdicts, inviting explanations, returning to dissenting jurors, and checking for consensus. Jury 2 provides the clearest example: the foreperson conducted an initial vote, identified a four-to-two split, and then invited the dissenting jurors to explain their reasoning before asking the guilty voters to do the same. Later, the foreperson returned to the group to ask whether anyone wished to change their verdict after hearing both sides. Other forepersons combined position-taking with facilitation. In Jury 5, the foreperson opened by announcing a guilty verdict but later asked the confederate to explain the basis for her uncertainty and entertained competing interpretations of the therapist’s professional status and obligations. Foreperson leadership, then, was not necessarily neutral; instead, it often involved expressing a personal position while simultaneously organizing and sustaining the group’s discussion.

Importantly, exchanges involving dissent often included substantive responses even when the disagreement did not correspond with differences in the final jury verdict. In Jury 1, the confederate questioned whether the child’s appearance alone provided sufficient evidence of abuse. Other jurors responded by returning to reasonable suspicion and the absence of a penalty for making a good-faith report—information provided in the jury instructions—after which the confederate accepted the group’s reasoning. A similar pattern emerged in Jury 3, where the confederate raised uncertainty about the former patient’s treatment history and the amount of information missing from the vignette. Rather than simply dismissing the concern, the foreperson and other jurors acknowledged the ambiguity before explaining why the available warning signs nevertheless justified reporting.

Jury 4 reflected a similar process. After the confederate suggested that the child’s disheveled and unkempt appearance could have been explained by camping in the wilderness and that the former patient’s treatment history was unknown, the foreperson acknowledged the plausibility of the argument before returning to the wording of the vignette and the patient’s prior inappropriate “feelings and impulses” around children. In this way, engagement with dissent often involved acknowledging an alternative interpretation, evaluating it, and then explaining why the original position was retained.

The one jury in which dissent emerged organically produced the most extended direct exchange between competing verdict positions. In Jury 2, a non-confederate juror initially voted not guilty and defended that position by emphasizing the ambiguity of the evidence and questioning whether the therapist could reasonably generalize from the former patient’s history. After hearing the competing arguments, the juror qualified the circumstances under which a guilty verdict would be appropriate and ultimately changed their vote to guilty. This jury also deliberated for 16 min, the longest session in the study. Given that only one jury exhibited organic dissent, however, this difference in duration should not be interpreted inferentially.

Taken together, the qualitative findings suggest that dissent was most visible not in differences in final jury outcomes, but in the conversational work that followed disagreement. Jurors clarified why particular evidence mattered, acknowledged ambiguity, responded to competing interpretations, and, at times, reformulated their reasoning before reaching consensus. These patterns provide additional context for the quantitative findings by illustrating forms of deliberative engagement that were not apparent from the final jury-level verdict alone.

## 4. Discussion

The present mixed-methods pilot study examined how individual juror characteristics, group discussion, and dissent operated across seven virtual mock-jury deliberations. Overall, the findings suggest that the interpersonal dynamics of deliberation may be particularly important to understanding how jurors participate and ultimately reach consensus. In preliminary exploratory analyses, collaborative conflict management showed a positive individual association with self-reported participation. In contrast, openness, agreeableness, the competing conflict style, and civic engagement were not associated with participation. Individual verdict preferences also shifted following deliberation, with all six participants who initially voted not guilty ultimately reporting a guilty verdict. Finally, although dissent emerged organically in only one jury, the observational findings suggest that disagreement—whether organic or introduced by the confederate—created opportunities for jurors to articulate competing interpretations of the evidence before reaching consensus. These findings are preliminary, given the small number of juries, but they nevertheless highlight an important distinction between the outcome of deliberation and the processes by which jurors arrive at it.

### 4.1. Individual-Level Differences and Deliberative Participation

Of the individual characteristics examined, collaborative conflict-management style was the only individual predictor significantly associated with self-reported participation, offering tentative, partial support for H1. However, because the full predictor model did not significantly improve fit over the unconditional model and random-intercept variance was estimated at zero, this finding should be interpreted strictly as an exploratory association. Rather than establishing a definitive predictive effect, these preliminary results suggest a potential link between jurors’ approaches to interpersonal conflict and their self-reported engagement during deliberation.

Interestingly, openness and agreeableness were not significantly associated with participation. This finding runs counter to expectations that more open-minded or agreeable jurors would be particularly receptive to discussion and negotiation with others (Olczak et al., 1991). Civic engagement was similarly unrelated to participation. One possibility is that broad personality characteristics and civic orientations do not translate directly into behavior once jurors enter an active group setting. Instead, a construct more directly tied to navigating interpersonal disagreement, such as collaborative conflict management, may be a candidate for further exploration in fully powered studies. That said, these null findings should be interpreted cautiously. With only 35 participants across seven juries, the present analyses had limited power to detect smaller associations, and the lack of significant effects should not be taken to suggest that personality or civic orientations are irrelevant to juror decision-making (Bornstein et al., 2017).

The observational findings provide some context for this interpretation. Participation across the seven deliberations was not simply a matter of how often jurors spoke. Jurors asked one another questions, clarified their interpretations of the vignette, responded to competing arguments, and, at times, acknowledged uncertainty before explaining why they ultimately maintained a particular position. Forepersons similarly varied in the extent to which they facilitated these exchanges by soliciting initial opinions, returning to dissenting jurors, and checking whether perspectives had changed. These patterns are broadly consistent with prior research emphasizing the importance of facilitation and interpersonal exchange to deliberative quality (Gastil et al., 2007). Furthermore, because individual conflict-management scores were not linked to specific conversational behaviors in the observational coding—and given the overall model fit limitations—these findings cannot establish that a collaborative conflict style produced these behaviors. Rather, they demonstrate the kinds of interaction that self-reported participation may reflect in active deliberation.

### 4.2. Verdict Change, Social Influence, and Dissent

Although verdict preferences did not shift toward the confederate’s dissenting position as predicted by H2, individual verdict preferences nevertheless changed following deliberation. All six participants who initially selected not guilty changed their verdict to guilty, while all participants with complete paired data who initially selected guilty maintained their verdict. This finding aligns with a substantial body of jury and group decision-making research demonstrating that individual judgments can shift as they are expressed and reconsidered within a group setting (Devine et al., 2001; Salerno & Diamond, 2010; Waters & Hans, 2009). The observed pattern is also consistent with classic conformity research demonstrating the influence that a clear majority can exert over individual group members (Asch, 1951, 1956). However, these findings cannot establish whether participants changed their verdicts in response to majority pressure, reconsideration of the evidence, or a combination of these influences.

Participants’ self-reported perceptions of verdict decision-making provide additional context for these findings. Although nearly all participants rated case evidence as very influential, perceptions of group discussion were more varied, with approximately three-quarters of respondents describing it as at least somewhat influential. Notably, most participants reported reaching their verdict when they first encountered the case materials, suggesting that initial judgments may have remained important throughout deliberation. However, these measures were collected retrospectively, and participants’ reported decision timing or perceived influences may not fully capture the processes underlying their final verdict preferences. Accordingly, the findings cannot determine whether the observed changes in verdicts reflected evidentiary reconsideration, social influence, or a combination of both.

Importantly, however, the present findings also demonstrate why verdict change alone provides an incomplete measure of conformity. Jury deliberation is specifically intended to expose jurors to alternative interpretations of the evidence, and a changed verdict may therefore reflect thoughtful reconsideration rather than simple accommodation to majority pressure (Austen-Smith & Banks, 1996; Gastil et al., 2007). At the same time, unanimity requirements and public voting create conditions in which normative pressure may operate alongside genuine persuasion (Asch, 1956; Devine et al., 2001). The present design cannot disentangle these processes. Thus, while the six not-guilty-to-guilty transitions clearly demonstrate that individual judgments changed following discussion, they cannot tell us *why* those judgments changed.

In this vein, the observational findings are particularly informative. Jurors did not always respond to disagreement by immediately abandoning or defending their position. Instead, they sometimes acknowledged that another interpretation was reasonable, identified ambiguity in the evidence, and then explained why they nevertheless reached a different conclusion. This pattern is consistent with the broader minority-influence literature, which suggests that exposure to dissent can encourage greater consideration of information, even when the minority position ultimately does not prevail (Asch, 1956; Bergold & Bull Kovera, 2022; Levine, 1999). In other words, the relevance of dissent may lie not only in whether it changes the final vote, but also in whether it encourages jurors to make their reasoning explicit. This distinction is especially important in the present study because every jury ultimately returned a guilty verdict. On the surface, this degree of consensus might suggest little variation across groups. The transcripts, however, tell a more complicated story. Jurors drew on factual and legal considerations—including reasonable suspicion, mandated-reporting obligations, and the therapist’s knowledge of the former patient—while also invoking moral judgments about professional responsibility, the child’s vulnerability, and the consequences of failing to intervene. Rather than representing wholly separate modes of decision-making, these considerations often appeared together as jurors worked through the case’s ambiguity. This finding is consistent with research suggesting that jurors construct and evaluate narratives from the available evidence rather than treating individual pieces of information in isolation (N. Pennington & Hastie, 1992; Salerno & Diamond, 2010).

### 4.3. Organic and Confederate-Introduced Dissent

H3 anticipated differences in deliberation length depending on how dissent emerged. The six juries in which the confederate introduced a dissenting position deliberated for an average of 9.17 min, whereas the one jury containing organic dissent deliberated for 16 min. Interestingly, the longest deliberation was therefore observed in the organically dissenting jury. Because organic dissent occurred in only one jury, however, this pattern cannot be evaluated inferentially and should not be taken as evidence that organic dissent necessarily produces longer or more substantive deliberation. This descriptive pattern is broadly consistent with research suggesting that pre-discussion disagreement can facilitate more intensive group discussion (Schulz-Hardt et al., 2006). However, discussion intensity and deliberation duration represent distinct aspects of group decision-making, and the present study cannot determine whether the longer deliberation reflected greater substantive engagement or other group processes.

Still, the distinction between organic and introduced dissent raises an important question for future jury research. Minority-influence research has long demonstrated that even a single nonconforming opinion can alter the social context of group judgment and encourage greater engagement with alternative perspectives (Asch, 1956; Bergold & Bull Kovera, 2022). The present deliberations provide a small-scale illustration of what this process may look like in practice. In the organically dissenting jury, the foreperson invited both sides to explain their reasoning before the group revisited its votes. In several juries in which early consensus emerged, the confederate’s alternative position similarly prompted jurors to return to the evidence and articulate why they believed the reporting threshold had or had not been met.

That said, the source of dissent was not experimentally manipulated. Every jury included a confederate, and the confederate introduced a dissenting position only when substantive disagreement had not already emerged. As a result, the organically dissenting jury may have differed from the other groups in ways that existed before the confederate ever spoke. The present study, therefore, cannot determine whether introduced dissent caused greater consideration of alternative perspectives or affected deliberation length. Rather, these findings point to a potentially important distinction for future work: whether disagreement that develops naturally among jurors functions differently from dissent deliberately introduced into an otherwise converging group. Taken together, the quantitative and observational findings suggest that the deliberative process may be consequential even when the ultimate verdict is not altered. Across these juries, disagreement created opportunities for jurors to clarify the basis of their positions, respond to alternative interpretations, and negotiate areas of uncertainty before reaching consensus. This is consistent with prior work suggesting that deliberation quality cannot be reduced solely to the verdict ultimately reached (Gastil et al., 2007; Waters & Hans, 2009). Particularly in unanimous decision-making environments, attending to how jurors reach agreement may therefore provide information that the final vote alone cannot capture.

### 4.4. Study Limitations and Future Directions

Several limitations should be considered when interpreting these findings, especially given the preliminary and largely exploratory nature of the present pilot study. The sample included only 35 non-confederate participants nested within seven juries. This modest sample, and especially the small number of jury-level clusters, limits the precision and reliability of the multilevel estimates (Bornstein et al., 2017). Indeed, the estimated jury-level variance in the participation model was zero (resulting in a singular model fit), and the full predictor model did not significantly improve fit over the unconditional model. Consequently, the statistical significance of the individual coefficient for collaboration style should be interpreted with caution. This result serves as a preliminary, exploratory observation rather than a robust predictive finding and should be replicated with larger samples, as well as with variations to the study design.

Additionally, the design does not permit a causal test of dissent. A confederate was physically present in every jury, and whether she introduced a dissenting position depended on whether disagreement had already emerged among the other jurors. There was no randomized dissent condition and no confederate-absent control group. Accordingly, the present findings cannot establish whether confederate-introduced dissent increased engagement, changed verdicts, or altered deliberation length. Further, because organic dissent occurred in only one jury, comparisons based on the source of dissent remain descriptive.

Further, the analysis of pre- to post-deliberation verdict shifts (H2) is constrained by non-independence. Individual jurors deliberated within seven discrete jury clusters, meaning individual pre-to-post vote shifts were nested within group interaction dynamics. While an exact McNemar test showed a significant overall shift toward Guilty (*p* = 0.031), the small number of jury clusters (*J* = 7) precluded the use of clustered McNemar tests or multilevel logistic models for dependent binary pairs. As reported in Table 5, the six Not Guilty-to-Guilty transitions were distributed across five separate juries. Future research with a larger sample of jury clusters should employ clustered categorical methods or multilevel modeling to explicitly account for intra-cluster correlation during group deliberation.

The deliberations were also relatively brief, ranging from 6 to 16 min. Although these times were taken directly from the recordings, the sessions were considerably shorter and less complex than deliberations following an actual criminal trial. Participants evaluated a written vignette rather than a full evidentiary record involving witnesses, exhibits, competing attorneys, and extended jury instructions, a limitation inherent in vignette-based psycho-legal research (Bieneck, 2009). Thus, the relatively brief discussions may reflect the more limited nature of the decision-making task rather than the length or complexity of deliberations that occur in real-world jury settings.

An additional limitation of the present study concerns the measurement of deliberative participation, which relied on a single self-reported item. While self-reports offer insight into participants’ subjective engagement, perceived participation may not fully align with or capture all dimensions of observable deliberative behavior (e.g., floor time, argument frequency, or back-and-forth exchanges). Further, reliance on a self-report measure to assess individual deliberation participation may have been affected by recall bias or subjective social comparison. While group-level deliberation duration was objectively recorded, individual-level verbal contributions were not separately tracked by speaker. Future studies evaluating jury dissent should incorporate objective individual behavioral measures, such as automated timestamped speaking durations.

To gain insight into whether verdict changes reflected evidence re-evaluation or social conformity, post-deliberation surveys also assessed participants’ perceived drivers of influence. Participants evaluated the degree to which their final verdict was shaped by case evidence versus overall group discussion and specific speakers. While these self-report items provide valuable pilot insights into participants’ conscious decision-making drivers, self-perceptions may not fully isolate subconscious normative compliance from genuine informational influence. Future studies should complement these self-report metrics with multi-wave longitudinal follow-ups (e.g., re-assessing verdicts weeks later) or implicit thought-listing protocols (Hafdahl et al., 2021) to evaluate the long-term persistence of evidence-based persuasion versus temporary social compliance.

Regarding the sample, participants were university students and are unlikely to be fully generalizable to jury-eligible community populations. Future studies in this area should use more diverse samples. The case materials also produced a pronounced tendency toward guilt: 29 of 35 participants initially selected guilty, and every jury ultimately reached a guilty verdict. This imbalance reduced opportunities for naturally occurring disagreement and may help explain why the confederate was required to introduce dissent in six of the seven groups. Future studies would benefit from pretesting case materials to produce greater initial verdict ambiguity.

The nature of the case materials also represents an important limitation. Although the vignette was selected to create ambiguity regarding the defendant’s responsibility for failing to report suspected abuse, the underlying allegations involved child sexual abuse and death and were therefore highly emotionally and morally salient. These features may have elicited strong reactions that influenced participants’ initial judgments, receptiveness to competing interpretations, or willingness to reconsider their verdict preferences during deliberation. Accordingly, the observed deliberative patterns cannot be assumed to generalize to cases involving less emotionally salient allegations. Future research should pretest case materials for both verdict distribution and emotional salience and replicate the present design using less evocative case scenarios.

All transcript coding was conducted by a single researcher, meaning interrater reliability could not be assessed. Although the content analysis provides descriptive insight into forms of argumentation and participation that the quantitative measures alone could not capture, the resulting categories necessarily reflect one researcher’s interpretation of the discussions. Future research should employ multiple independent coders, establish explicit coding criteria, and assess interrater agreement.

While the present study examined individual-difference constructs in relation to overall participation and verdict shifts, an important question remains regarding how these traits shape the qualitative substance of deliberation following dissent. Conceptually, certain traits, such as high openness or collaborative conflict preferences, may serve as psychological buffers during discussions, encouraging jurors to engage constructively with dissenting arguments rather than dismissing nonconforming views immediately. As qualitative deliberation coding in this pilot was conducted at the aggregate group level without speaker-specific survey linkage, we could not empirically match individual trait scores to specific qualitative dialogue themes. Future jury research should build on this work by using direct participant-linked qualitative coding to test whether specific individual traits directly predict constructive engagement versus resistance themes during deliberation.

Lastly, the study was conducted entirely through Zoom and included no in-person comparison condition. The findings, therefore, cannot establish whether any of the observed patterns resulted from virtual deliberation or would differ in a face-to-face setting. Videoconferencing may shape conversational turn-taking, social presence, attention, and other features of group interaction, making modality an important direction for continued research. In the present study, however, the virtual format should be understood as the setting in which deliberation occurred rather than as an experimentally tested explanation for the findings.

## 5. Conclusions

Despite these limitations, the present pilot study illustrates the value of examining jury decision-making at multiple levels. Verdict change captured whether individual preferences shifted following discussion, self-reported participation captured one dimension of juror engagement, and transcript analysis revealed features of reasoning and interaction that neither measure alone could represent. In particular, the findings suggest that disagreement may remain consequential even when it does not produce variation in the final group verdict. Jurors responded to competing interpretations, clarified the evidentiary and legal bases of their positions, acknowledged ambiguity, and negotiated consensus throughout discussion. The findings also provide tentative, exploratory grounds for further investigation into whether collaborative approaches to interpersonal conflict are associated with participation in jury deliberation. Future research with larger numbers of juries, experimentally manipulated dissent, more balanced case materials, multiple qualitative coders, and both virtual and in-person conditions will be necessary to determine when and how these processes shape jury decision-making. The present findings emphasize a broader point: understanding jury deliberation requires attention not only to what verdict a group ultimately reaches, but also to how jurors engage with disagreement on the way to that outcome.

## Figures and Tables

**Table 1:** Participant Demographic Characteristics.

Characteristic	*n*	%
Gender		
Female	28	80.0
Male	7	20.0
Race		
White	29	82.9
Black	4	11.4
Asian	1	2.9
White and American Native	1	2.9
Hispanic or Latino		
Neither	34	97.1
Both	1	2.9
Income		
$15,000–$24,999	1	2.9
$25,000–$34,999	1	2.9
$35,000–$49,999	1	2.9
$50,000–$74,000	7	20.0
$75,000–$99,000	7	20.0
$100,000–$149,999	9	25.7
$150,000–$199,999	5	14.3
$200,000 and above	3	8.6
Age		
18–20	27	77.1
21–44	8	22.9
Major		
Natural Sciences	3	8.6
English	2	5.7
Social Sciences	12	34.3
Engineering	5	14.3
Economics/Business	4	11.4
Other	9	25.7

Note. *N* = 35. Percentages may not total 100 due to rounding. Demographic category labels reflect the response categories reported in the original table.

**Table 2:** Descriptive Statistics and Internal Consistency for H1 Study Variables.

Variable	M	SD	Observed Range	α
Open-Mindedness	3.48	0.76	1.67–4.67	0.77
Agreeableness	3.95	0.65	2.00–4.83	0.78
Competing Conflict Style	3.32	0.78	2.00–5.00	0.70
Collaborating Conflict Style	3.86	0.70	1.33–5.00	0.76
Civic Engagement	18.66	3.83	7–26	—
Deliberative Participation	1.74	0.70	1–3	—

Note. *N* = 35. Open-Mindedness and Agreeableness scores were calculated as the mean of six items rated from 1 to 5. Competing and Collaborating conflict-management scores were calculated as the mean of three items rated from 1 to 5. Civic Engagement represents a composite index. Deliberative Participation was assessed using a single post-deliberation item ranging from 0 (none at all) to 3 (a great deal). Internal consistency is not reported for Civic Engagement because it was treated as a composite index, or for Deliberative Participation because it was assessed with a single item. α = Cronbach’s alpha.

**Table 3:** Multilevel Linear Model Predicting Self-Reported Deliberative Participation.

Predictor	b	SE	95% CI	*t*	*p*
Intercept	1.065	1.374	[−1.629, 3.759]	0.78	0.444
Open-Mindedness	−0.003	0.150	[−0.298, 0.292]	−0.02	0.983
Agreeableness	−0.098	0.214	[−0.518, 0.321]	−0.46	0.649
Competing	0.078	0.165	[−0.246, 0.402]	0.47	0.638
Collaborating	0.398	0.181	[0.043, 0.753]	2.20	0.035
Civic Engagement	−0.038	0.029	[−0.095, 0.018]	−1.34	0.188

Note. *N* = 35 participants nested within seven juries. Estimates are unstandardized coefficients from a linear mixed-effects model estimated using maximum likelihood. Degrees of freedom and significance tests for fixed effects were calculated using Satterthwaite’s approximation. Jury-level random-intercept variance was estimated as 0.000 and residual variance as 0.392 (ICC = 0.00), resulting in a singular model fit. The full model did not significantly improve fit relative to the unconditional model, χ^2^(5) = 6.88, *p* = 0.230. CI = confidence interval.

**Table 4:** Pre- to Post-Deliberation Verdict Transitions.

Pre-Deliberation Verdict	Post: Not Guilty	Post: Guilty	Total
Not Guilty	0	6	6
Guilty	0	28	28
Total	0	34	34

Note. *N* = 34 participants with complete pre- and post-deliberation verdict data. Six participants (17.6%) changed their verdict following deliberation; all changes were from not guilty to guilty. An exact two-sided McNemar test indicated a significant difference between pre- and post-deliberation verdicts (*p* = 0.031).

**Table 5:** Jury-Level Distribution of Pre- and Post-Deliberation Verdicts and Verdict Shifts.

Jury	Pre (G/NG)	Post (G/NG)	Missing Post	Verdict Shifts	Length (min)	Confed. Dissent	Organic Dissent	Group Verdict
1	5/0	5/0	0	0	6	Yes	No	Guilty
2	4/1	5/0	0	1	16	No	Yes	Guilty
3	3/2	5/0	0	2	10	Yes	No	Guilty
4	4/1	5/0	0	1	8	Yes	No	Guilty
5	4/1	4/0	1	1	11	Yes	No	Guilty
6	5/0	5/0	0	0	12	Yes	No	Guilty
7	4/1	5/0	0	1	8	Yes	No	Guilty
Total	29/6	34/0	1	6	—	6 Yes	1 Yes	7 Guilty

Note. G = guilty; NG = not guilty. The sample included 35 participants across seven mock juries, excluding confederates. Pre-deliberation verdicts were available for all 35 participants; post-deliberation verdicts were available for 34 participants. One participant in Jury 5 did not provide a post-deliberation verdict. Individual verdict shifts represent changes from participants’ pre- to post-deliberation survey responses, whereas final group verdicts represent the collective decisions reached during deliberation. Confederate-introduced dissent refers to disagreement introduced by the confederate, whereas organic dissent refers to a non-confederate juror expressing a dissenting position before the confederate’s initial vote. All seven juries reached collective guilty verdicts.

## Data Availability

The data presented in this study are available upon request.

## Supplementary Materials

The following supporting information can be downloaded at: https://www.mdpi.com/article/10.3390/bs16101960/s1.

## Appendix A. The Vignette

A therapist, who is a mandated reporter, is holding a nature retreat for clients in Little River Canyon, which is a National Preserve in Alabama. While hiking in the canyon with her group, she notices a young girl and a much older adult man walking together in front of her. As she approaches, she realizes that the older man is a former patient of hers who, at the time of counseling five years prior, struggled with inappropriate feelings and impulses around underage children. When looking at the child, she appears to be thin, unshowered, and visibly upset. As the older man recognizes his former therapist, he seems nervous and holds the child close to him. The therapist is concerned about this scene but does not immediately spring into action, as she is currently leading a nature retreat for her clients and has not actively worked with this client in several years. Instead, she walks closer to them and asks the man: “Is this your little one?” to which the child looks at her scared. After a pause, the man says “Yes, this is my granddaughter! Say hi, Margie!” The child does not respond or look up at the therapist. Afterward, the therapist, still feeling a bit uneasy but still unsure of the scene, continued walking past the pair.

Two days later, as the therapist and her clients are packing up to leave the campsite, she is approached by park police asking her if she has seen an older man with a much younger girl. She says yes, and the police ask to take her statement. It is then that she learns that little girl was not, in fact, related to her former patient and was a victim of child abuse and is now deceased.

Failure to report as a mandated reporter is a federal civil AND criminal charge. In this case, imagine that only criminal charges (guilty/not guilty) are being put forth.

Do you think that the therapist should be found guilty of the crime of failure to report?

Federal conditions for mandatory reporting of child abuse:

A person who, while engaged in a professional capacity or activity on Federal land or in a federally operated (or contracted) facility, learns of facts that give reason to suspect that a child has suffered an incident of child abuse, and fails to make a timely report, shall be fined under this title or imprisoned not more than 1 year or both. (Duty to Report Suspected Child Abuse Under 42 U.S.C. § 13031)

## Appendix B. Jury Instructions (Condensed)

Statement of Charge

The therapist, the defendant in this case, has been accused of the crime of failing to report child abuse as a mandated reporter.

Please note that this crime took place on federal land and thus is handled in federal criminal court.

While giving these instructions, I will act as the “Judge” in the case. If you have any questions related to the instructions, please wait until after I give these instructions and for deliberation to begin. At that time, I will act as the “bailiff” and while I will not be able to assist you in your understanding of these instructions nor with the content of the case itself, the foreperson may submit any written questions for the Judge in the Zoom chat.

*To select a foreperson, you must elect or identify a person in your group who will act as the spokesperson for the jury.*

For a federal criminal charge of failure to report, all following conditions must be proved beyond a reasonable doubt:

(1) Persons engaged in the following professions and activities are subject to mandatory reporting requirements:
  (a) Physicians, dentists, hospital personnel and other health care workers.
  (b) Psychologists, psychiatrists, and mental health professionals
  (c) Social workers, licensed or unlicensed marriage, family, and individual counselors.
(2) The mandated reporter knows, or has reasonable suspicion, that—
  (a) a child was abused in federal lands,
  (b) actions are being taken, or are going to be taken, that would reasonably be expected to result in abuse of a child on federal lands.
(3) The mandated reporter fails to immediately report such abuse or actions to the local child protective services agency or local law enforcement agency, shall be fined under this title or imprisoned for not more than 1 year or both.

Any person making a report which is based upon their reasonable belief, and which is made in good faith shall be immune from civil or criminal liability for making that report.

The therapist can still be charged with failure to report even if:

(a) She did not possess prior knowledge of the child nor his/her identity
(b) The child resided or was cared for on federal lands at the time of abuse and her eventual death

The therapist cannot have criminal charges brought against her if:

(a) She had no awareness of her duty to report nor the criminal implications of failing to do so.

If you unanimously conclude that the above conditions cannot be proven beyond a reasonable doubt, then you must render a verdict of “not guilty.”

Only one verdict may be returned. The verdict must be unanimous, meaning everyone must agree. Failure to come to a unanimous vote will result in a hung jury or a mistrial.
