# Supplementary material for: The Role of the Dissenting Voter on Jury Deliberation Online: A Pilot Study
Source: Behav Sci (Basel). 2026 Oct 21;16(10):1960. doi: 10.3390/bs16101960 (PMC13701490; doi:10.3390/bs16101960)
Supplement: Supplementary file 1 [file behavsci-16-01960-s001.zip › behavsci-4567590-supplementary.pdf]

Confederate Script

The confederate will enter the Zoom room at the same time that all participants are due to arrive and will behave as a participant in the focus group. Her demeanor will be kind and cordial, but not overly kind, or sociable, nor will she be overly aloof or disagreeable.

The facilitator will read aloud the vignette and jury instructions, making it clear that the group must come to a unanimous decision, which will be indicated by a verbal "yes". Time will then be devoted to the selection of a foreperson. **The confederate will NOT be allowed to be the foreperson. If someone selects her, she can kindly decline.** If no one can select a foreperson, the facilitator will at random.

The facilitator will then explain that the foreperson should check in with everyone about their votes at the beginning, in the middle of discussion, and then at the end for me (the Judge) to record. These **check-ins need to be verbal in nature**, so that our confederate can keep track of the number of votes cast.

Once the vignette is presented and the **first majority opinion** is expressed, the confederate will chime in with her thoughts/opinions that, regardless of personal feeling, will be the opposite view of the one expressed. **PLEASE NOTE THAT WHILE THE CONFEDERATE WILL ATTEND EVERY MEETING, SHE WILL NOT ALWAYS NEED TO EXPRESS AN OPPOSITIONAL OPINION. SEE QUALIFIERS BELOW FOR MORE INFORMATION.**

In order to quantify what the "first majority opinion looks," we define it as follows: the first opinion that is verbally "yes/no/agree/not guilty/guilty" shared by at least 75% of members in the Zoom, outside of the facilitator ***but including the confederate*** (based on Davenport & Winet, 2022). For example, if we obtain the ideal number of 6 jurors, 5 of 6 would have to unanimously agree, without any debate **(see Scenario 4)** before the confederate jumps in.

Counts to remember:

If 6 members, 5 members in agreement (75% is 4.5, so we round up)

Qualifiers to Confederate participation:

Scenario 1: **The deciding/discussion phase**

IF the "first majority opinion" is not expressed within the first 10 minutes of the group discussion, then the confederate will posit:

"Well, what are people thinking?" Then, based on those responses, she will voice her opposing views **after the first majority vote, if applicable.**

IF someone then asks the confederate: "I don't know, how about you?" **she will say "I think I need to hear other people's opinions first before I can fully express my own."**

Scenario 2: **Noncompliance/No talking amongst group (rare)**

IF, in the rare case that the group is at a standstill and no progress has been made, **facilitator** will initiate by saying the following:

"I think we are at a standstill. Does it not appear as though a decision can be reached by the group?"

Scenario 3: **Immediate disagreement/nonconformity**

IF participants immediately start debating upon hearing jury instructions and the selection of the foreperson, then our confederate will operate as a normal participant during deliberation processes.

“Normal participation” will be defined as participating in conversation organically, being able to express opinions openly and honestly.

Examples of nonconformity without the need for a confederate:

-Right at the top, some jurors agree, and some disagree and organic discussion ensues

-At first some people conform, but someone breaks conformity BEFORE the 5th person.

-breaking conformity can look like any of the following (and variations of it):

- -agreement with some aspects of an argument, but introduction of a new issue
- -overall disagreement
- -disagreement on technical aspects (i.e. jury instructions, burden of proof, evidence provided)
- -agreement at first and then a change of mind before the 5th conforming vote

When participating as a normal juror, CONFEDERATE can vote whenever she pleases (she does not need to be the final voter)

Scenario 4: Immediate agreement/conformity

IF after the jury instructions are given and the foreperson is selected jury members immediately express the same opinion WITHOUT further debate, qualifiers, or discussion, the confederate will interject after the 5th person has expressed a conforming opinion, using either the script examples below or a variation of the script.

Examples of conformity without further discussion or debate:

-Jurors 1-6 agree **WITHOUT** any “but what if we also consider...”

-Some jurors aren’t as confident but do not bring up any counterpoints or arguments

- Jurors ask clarifying questions but do not posit another route of discussion (i.e. “was the person the man with underage?”)

-Jurors bring up a potential counterargument **BUT THE POINT IS IGNORED and conformity continues (rare)**

I want this script to be loose, so CONFEDERATE has creative liberties to produce reason as to why she disagrees with the group decision, but **I also want the points of disagreement to adhere to the vignette** (i.e. please do not say “therapists have too much on their plates these days to keep track of everything” or “Old men are so creepy”; Try to keep points relevant to the content of the script).

WHEN the foreperson is counting his/her/their final vote, the confederate should ALWAYS be the last person to vote. If the foreperson asks her earlier, say “come back to me, please.”

In this final vote, CONFEDERATE will cast her vote in accordance with whatever the new majority opinion is (even if it is different from her original counterargument).

TOPICS FOR CONFEDERATE TO MENTION WHEN INTERJECTING AS THE CONFEDERATE:

Points that confederate could mention **if group FINDS the therapist guilty**:

-therapist was working with a separate group. How should she be expected to direct her attention to another situation entirely?

-she used her best discretion

-she had not worked with client in five years

-children can be fussy/messy/dirty, especially while camping
-perhaps the man looked nervous because he recognized his therapist in public
-perhaps the man looked nervous because he recognized a person in the therapy retreat group
-perhaps he was seeking therapy during a dark period of his life that no longer applies to him
-the vignette says nothing about visible wounds on the child, nor defensive wounds on the man
-the vignette says nothing about the man ever acting on his impulses in the past

---

Points that confederate could mention **if group FAILS TO FIND the therapist guilty:**
-the therapist is still a mandated reporter, regardless of working with another client/group of clients.
-the therapist was on duty.
-the therapist had prior knowledge of the man's struggles with pedophilia
-even though it had been a few years, the man was unsupervised with a child and the child appeared upset
-even though the therapist still admitted to feeling "uneasy" after her interaction with the pair, she did nothing
-she stopped to inquire about their situation and still did nothing
-there is no penalty for reporting just in case
